# Glucose-6-Phosphate Dehydrogenase Activity and Protein Oxidative Modification in Patients with Type 2 Diabetes Mellitus

**DOI:** 10.1155/2013/430813

**Published:** 2013-12-22

**Authors:** Aida A. Mahmoud, Amal K. A. Nor El-Din

**Affiliations:** ^1^Biochemistry Department, Faculty of Medicine, Sohag University, Sohag, Egypt; ^2^Internal Medicine Department, Faculty of Medicine, Sohag University, Sohag, Egypt

## Abstract

*Objectives*. The aim of the present investigation was to study the activity of glucose-6-phosphate dehydrogenase (G6PD) and correlate its activity to protein oxidation markers in type 2 diabetic patients under poor glycemic control. *Methods*. G6PD activity, protein carbonyl group concentration, and total thiol group content were measured in blood samples of 40 patients with type 2 diabetes mellitus under poor glycemic control and 20 healthy control subjects. *Results*. G6PD activity and total thiol group content decreased significantly while glycated hemoglobin (HbA_1C_) and protein carbonyl group concentration increased significantly in diabetic patients than in the controls (*P* < 0.001). In addition, Obtained results revealed that, in diabetics, G6PD activity negatively correlated to protein carbonyl and HbA_1C_ (*r* = −0.77 and −0.65, resp.), while positively correlated to total thiol (*r* = 0.66) and protein carbonyl negatively correlated to total thiol (*r* = −0.85), while positively correlated to HbA_1C_ (*r* = 0.43). Also in controls, G6PD activity negatively correlated to protein carbonyl and HbA_1C_ (*r* = −0.57 and −0.56, resp.), while positively correlated to total thiol (*r* = 0.5) and protein carbonyl negatively correlated to total thiol (*r* = −0.48), while positively correlated to HbA_1C_ (*r* = 0.68). *Conclusions*. We concluded that G6PD activity decreased in diabetics than in controls and was negatively correlated to oxidative stress markers and HbA_1C_. G6PD activity can be taken as a biomarker of oxidative stress and poor glycemic control in type 2 diabetic patients.

## 1. Introduction

Diabetes mellitus is a metabolic disease characterized by hyperglycemia due to defects in insulin metabolism. If the hyperglycemia of diabetes is not managed properly, it causes long-term damage, dysfunction, and failure of different organs, notably the eyes, kidneys, nerves, heart, and blood vessels [1]. An increase in oxidative stress has been observed in diabetic patients, which may be due to an increase in processes that produce oxidants or due to a decrease in the antioxidant defense mechanisms [2].

Glucose-6-phosphate dehydrogenase (EC1.1.1.49; D-Glucose-6-phosphate: NADP^+^ oxidoreductase) is the rate-limiting enzyme of the pentose phosphate pathway. It is required for the antioxidant defense because it produces NADPH, the main cellular reductant and the fuel for glutathione recycling within the cells [3] G6PD plays a central role in cell metabolism and was found to play pathophysiologic roles in many diseases like diabetes, aldosterone-induced endothelial dysfunction, and cancer. The central role of G6PD is being the major source of NADPH, a hydrogen carrier required by many essential cellular systems like glutathione recycling, nitric oxide synthesis, cytochrome p450 system, and others [4]. There is increasing evidence that G6PD activity is of major importance for NADPH production for defense against oxidative stress rather than for ribose production [5]. It was found that hyperglycemia of diabetes mellitus and high glucose levels decrease the activity of G6PD and the inhibition was through the increase in adenylate cyclase activity which in turn increases cAMP levels within the cell [6].

Carbonyl (CO) groups are considered as the most general indicator of protein oxidation. CO groups (aldehyde and ketones) are produced on protein side chains (especially of Pro., Arg., Lys., and Thr.). When proteins are exposed to oxidants. Protein carbonyl derivatives are also generated when reducing sugars react with lysine residues of proteins with the eventual formation of advanced glycation end products such as pentosidine and carboxymethyllysine [7].

Thiol groups either in free form or bound to proteins play a major role in maintaining the antioxidant status, both intracellularly and extracellularly. Thiols are compounds which contain the sulfhydryl group (–SH) attached to a carbon atom. The reduced thiols found in human plasma are, homocysteine (HcySH), cysteinylglycine (CysGlySH), cysteine (CysSH), and glutathione (GSH) [8]. Albumin exists in both reduced and oxidized forms in systemic circulation and the reduced form of the human serum albumin has been shown to be lower in patients with hepatic disorders, diabetes, and renal diseases. The bulk of free thiol in plasma is represented by Cys-34 of albumin [9]. Thiols help cytoplasm of aerobic cells to maintain a reducing state in the presence of an oxidizing environment [10].

This investigation aimed to study G6PD activity as a biomarker of oxidative stress and correlate its activity to protein oxidation markers; protein carbonyl group concentration and total thiol group content in type 2 diabetes mellitus.

## 2. Materials and Methods

### 2.1. Patients and Controls

*Patients*. Forty patients (15 ♂ and 25 ♀) with type 2 diabetes mellitus, diagnosed according to the criteria of the Expert Committee [11], were recruited from the department of internal medicine, Sohag University Hospital. Exclusion criteria were, presence of diabetic complications, obesity (BMI ≥ 30), HbA_1C_ less than 7% and hemolytic disorders. The clinical characteristics of the patients were indicated in Table 1.

*Controls*. Twenty healthy control subjects, their age and sex distribution were similar to those of the patients. They were subjected to complete medical examination to exclude the presence of medical problems. They were recruited from the hospital staff.

Informed consent was taken from all the participants and all the experiments were strictly adhered to the tenets of the Helsinki declaration.

### 2.2. Blood Samples

All subjects were advised to take no medication on the morning before blood samples collection. Fasting blood (5 mL) was obtained from the antecubital vein, after an overnight fasting period (10–12 hours). Samples were divided into two parts: one containing trisodium citrate (final concentration 1 mg/mL) for the estimation of G6PD activity. Samples were centrifuged at 1,800 g for 10 minutes at 4°C, then, the plasma, buffy coat, and upper 15% of the packed RBCs were removed and the remaining RBCs were washed twice with PBS and lysed with 9 volumes hypotonic solution (5 mM phosphate buffer, PH 7.4) on ice. For the estimation of protein carbonyl and total thiol; 3 mL venous blood was taken into vacutainer clotted tubes and centrifuged at 1,800 g for 10 minutes and sera were frozen in aliquots and stored at −20°C till time of assay.

### 2.3. Chemicals

NADP (sodium salt), D-glucose-6-phosphate (monosodium salt), 6-Phosphogluconate (trisodium salt), Guanidine hydrochloride, and 5,5′-Dithiobis (2 Nitrobenzoic acid) were purchased from Sigma (St. Louis, MO, USA), and other chemicals were form MERK (Darmstadt, Germany).

### 2.4. Methods

Fasting blood glucose, lipid profile and HbA_1C_ were done for all the patients and controls using Cobas c 311 analyzer, Roche Diagnostics, Germany. G6PD activity assay was performed, using the fresh samples as described by Tian et al. [12]. In this method, the conversion of NADP^+^ to NADPH catalyzed by the two dehydrogenase enzymes in the pentose phosphate pathway was measured by the increase in absorbance at 340 nm due to the conversion of NADP^+^ to NADPH by either G6PD or by the second enzyme 6-phosphogluconate dehydrogenase (6PGD). G6PD catalyzes the conversion of glucose 6-phosphate to 6-phosphogluconolactone, which is rapidly hydrolyzed to 6-phosphogluconate (the substrate for PGD). Thus, to obtain accurate enzyme activities for G6PD and PGD, both PGD activity alone and total dehydrogenase activity (G6PD and PGD) were measured separately. G6PD activity was calculated by subtracting the activity of PGD from total enzyme activity. The unit of enzyme activity is defined as 1 *μ*mol of NADPH formed per minute per gram of hemoglobin. Protein carbonyls were measured according to the method of Reznick and Packer [13]. Thiol group was determined based on thiol/disulfide reaction of thiol and Ellman's reagent (5,5′-dithiobisnitrobenzoic acid) [14].

### 2.5. Statistical Analysis

Data were expressed as mean ± SD, the coefficient of the variation (CV%) of the studied parameters was calculated and data were analyzed using student's *t*-test and Pearson's correlation analysis. *P*-values lower than 0.05 were considered significant. Prism 3 version 5, http://www.Graphpad.com/ was used for analysis.

## 3. Results

**Figure 1:**
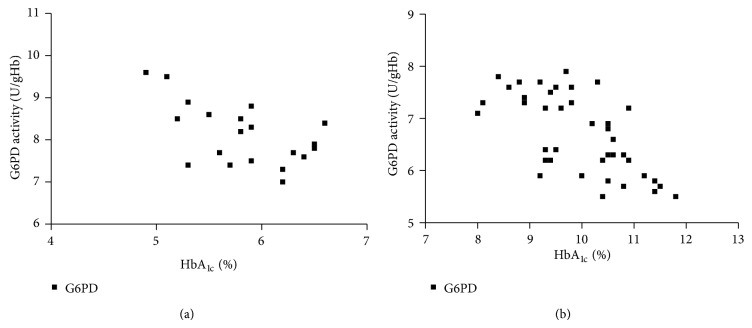
Correlation between HbA_1C_ and G6PD activity in controls (a) and diabetics (b).

**Figure 2:**
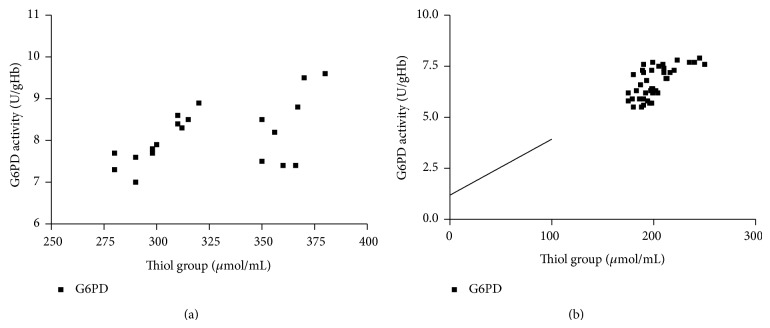
Correlation between thiol group and G6PD activity in controls (a) and diabetics (b).

**Figure 3:**
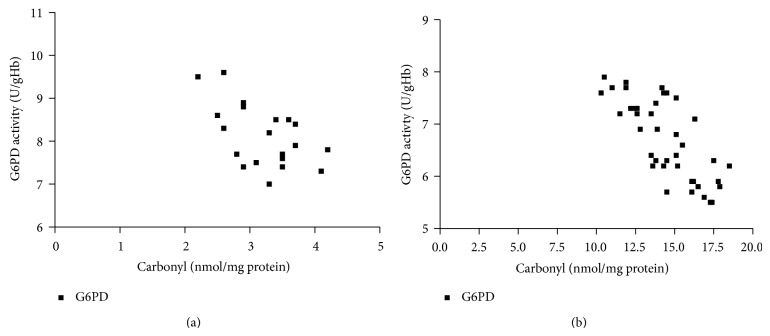
Correlation between carbonyl group and G6PD in controls (a) and diabetics (b).

**Figure 4:**
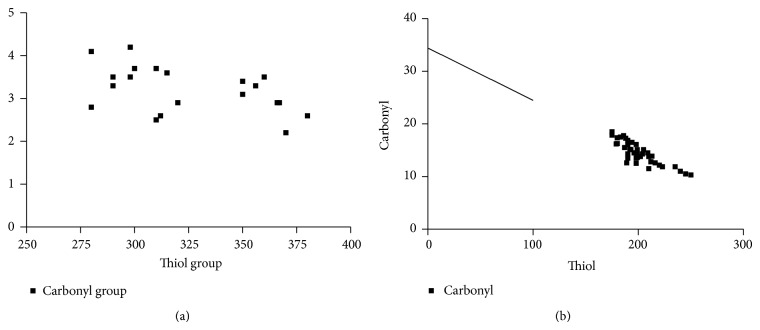
Correlation between carbonyl group and thiol group in controls (a) and diabetics (b).

**Figure 5:**
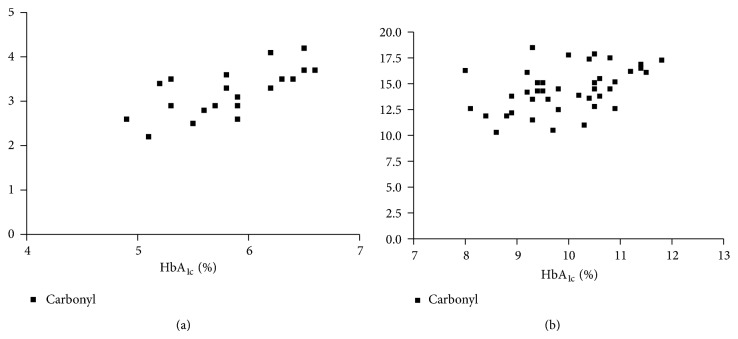
Correlation between HbA_1C_ and carbonyl group in controls (a) and diabetics (b).

**Figure 6:**
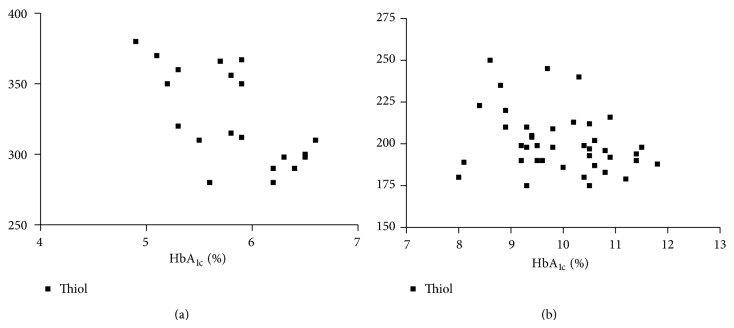
Correlation between HbA_1C_ and thiol group in controls (a) and diabetics (b).

The clinical and laboratory characteristics of the participants are represented in Table 1. Obtained results revealed that G6PD activity and total thiol group decreased significantly in diabetic patients than in the controls (*P* < 0.001), while carbonyl group content increased significantly in diabetics than in controls (*P* < 0.0001). In diabetic patients and controls, G6PD activity negatively correlated to protein carbonyl and HbA_1C_, while positively correlated to total thiol. In addition, protein carbonyl negatively correlated to total thiol, while positively correlated to HbA_1C_ (Table 2), (Figures 1, 2, 3, 4, 5, and 6), indicating the role of poor glycemic control in the development of oxidative stress and diabetic complications.

## 4. Discussion

In the present investigation, the effects of diabetes mellitus on the activity of G6PD and markers of protein oxidation, protein carbonyl group concentration, and total thiol group content in patients with type 2 diabetes mellitus under poor glycemic were studied. The investigation revealed that the activity of G6PD and total thiol levels decreased significantly in diabetic patients compared to the controls (*P* < 0.001), while protein carbonyl was significantly higher in diabetic patients than in controls (*P* < 0.001). In addition, G6PD activity negatively correlated to protein carbonyl and HbA_1C_, while positively correlated to total thiol and protein carbonyl negatively correlated to total thiol, while positively correlated to HbA_1C_. Hyperglycemia decreases G6PD activity, supported by experimental observations. Zang et al. [15] found that the culture of bovine endothelial aortic cells at high-glucose concentration, caused activation of protein kinase A which led to the phosphorylation of G6PD and to a decrease in its activity. The same results were observed in the kidney cortex of diabetic rats and were reversed with insulin treatment [16]. Both posttranslational mechanisms and decreased gene expression appear to be involved in the decrease of G6PD activity that was observed after exposure to high levels of hyperglycemia (20–30 mmol/L). Recently, it has been shown that high glucose also decreased G6PD expression and activity in human islets of pancreas [17]. Hyperglycemia may increase oxidative stress, which affects the normal function of cellular proteins and enzymes. High glucose level was found to activate adenylate cyclase which increases cAMP levels. cAMP activates protein kinase A, an inhibitor of G6PD. G6PD is the major source of NADPH, the main intracellular reductant; hence the decrease in its activity increases the oxidative stress and this was obvious in this investigation through the positive correlation between G6PD activity and protein carbonyl and the negative correlation between G6PD activity and total thiol [18]. In addition, hyperglycemia induces mitochondrial superoxide production which in turn activates four damaging pathways in cells through inhibiting the activity of the key glycolytic enzyme glyceraldehyde-3-phosphate dehydrogenase. Increased levels of glyceraldehyde-3-phosphate activate advanced glycation end product pathway, protein kinase C pathway, hexosamine pathway, and polyol pathway [19]. It is also found that hyperglycaemia induces effects within the cell nucleus through reactive oxygen species (ROS). Hyperglycaemia initiates a cascade of transcription events, ultimately leading to changes in the levels of NO, cytokines, acute-phase reactants, and cellular adhesion molecules. Generation of ROS can be reduced by avoiding hyperglycaemia and by minimizing fluctuations in blood glucose levels [20].

Several investigators found significant increase in protein carbonyl in diabetic patients [21–24]. The maintenance of protein redox status is essential for cell function, so structural changes in proteins are considered to be among the molecular mechanisms leading to diabetic complications. The introduction of CO groups into proteins causes alterations in protein conformations leading to increased aggregation, fragmentation, distortion of secondary and tertiary structure, and susceptibility to proteolysis, and decrease of normal function [25, 26].

Arif et al. and Çakatay, [21, 22] found decreased thiol levels in both type 1 and type 2 diabetes mellitus. The decreases can be due metabolic, inflammatory or iron alterations [27]. Free iron in the ferric state was found to be increased significantly in diabetic patients under poor glycemic control and this finding can explain the decrease in protein thiols [28]. Thiols were found as facile targets of glycation and low molecular mass thiols as potent glycation inhibitors. This finding may aid the design of therapeutic agents for the treatment of the complications of diabetes [29]. Uncontrolled hyperglycemia can lead to numerous pathological conditions, eventually resulting in long-term microvascular and macrovascular complications [1]. The antioxidant enzymes usually studied in diabetes include catalase, superoxide dismutase, glutathione system, and thioredoxin system. The novelty of this study was the evaluation of G6PD activity and the correlation of its level to protein oxidation markers. The limitation of this investigation is that a longitudinal study is required, in which measurement of G6PD activity in the same patient takes place in two occasions: under poor glycemic control and after proper management of diabetes to exclude inherited deficiency of the enzyme and emphasize the role of improper management of the disease in the decrease in G6PD activity.

## 5. Conclusion

Improper management of diabetes mellitus caused a decrease in G6PD activity and an increase in protein oxidation markers: protein carbonyl group and total thiol group. G6PD activity can be taken as a biomarker of oxidative stress and poor glycemic control in type 2 diabetes mellitus patients.

## Figures and Tables

**Table 1:** The clinical and laboratory characteristics of the participants.

Parameter	Control (*n* = 20)	Diabetics (*n* = 40)
Age (years)	58 ± 7.1	57 ± 7
♂/♀ ratio	8 : 12	15 : 25
Medications	—	Oral hypoglycemic drugs
BMI (kg/m^2^)	23.9 ± 2.1 (8.4)	23 ± 1.7 (7.2)
FBG (mg/dL)	101 ± 10.8 (10.7)	274 ± 50^***^ (18.4)
TC (mg/dL)	185 ± 13 (7.1)	238.5 ± 62.4^**^ (26.1)
HDL (mg/dL)	39 ± 3.8 (10.1)	30.5 ± 7.2^**^ (23.6)
LDL (mg/dL)	183 ± 13.7 (7.5)	229 ± 40^*^ (17.4)
Triglyceride (mg/dL)	166 ± 32 (19.5)	176.5 ± 57 (32.5)
HbA_1c_ (%)	5.8 ± 0.5 (8.68)	9.9 ± 0.96^***^ (9.7)
G6PD activity (U/gHb)	8.1 ± 0.7 (8.9)	6.7 ± 0.76^***^ (11.4)
Protein carbonyl (nmol/mg protein)	3.2 ± 0.54 (16.7)	14.5 ± 2.14^***^ (14.8)
Total thiol (*μ*mol/mL)	325 ± 33 (10.3)	201 ± 18^***^ (9.1)

Data were represented as mean ± SD (CV %). FBG: fasting blood glucose; TC: total cholesterol; HDL: high density lipoprotein; LDL: low density lipoprotein; ^***^ *P* < 0.001, ^**^ *P* < 0.01, ^*^ *P* < 0.05.

**Table 2:** Pearsons correlation between the parameters.

	Control	Diabetics
G6PD activity & HbA_1c_	−0.57∗∗	−0.65∗∗∗
G6PD activity & total thiol	0.5∗	0.66∗∗∗
G6PD activity & protein carbonyl	−0.56∗	−0.77∗∗∗
Protein carbonyl & total thiol	−0.48∗∗	−0.85∗∗∗
Protein carbonyl & HbA_1c_	0.68∗∗∗	0.43∗∗
Total thiol & HbA_1c_	−0.66∗∗	−0.29∗

^***^ *P* < 0.001, ^**^ *P* < 0.01, ^*^ *P* < 0.05.
